# Charting Africa’s digital public health future: Five priorities for action

**DOI:** 10.4102/jphia.v17i1.1857

**Published:** 2026-02-27

**Authors:** Ngashi Ngongo, Nebiyu Dereje, Shanelle Hall, Jean Kaseya

**Affiliations:** 1 Africa Centres for Disease Control and Prevention, Addis Ababa, Ethiopia

With a population projected to reach 1.7 billion by 2030, the African continent faces an unprecedented opportunity to harness digital innovation to accelerate universal health coverage, strengthen epidemic preparedness and advance Africa’s Health Security and Sovereignty (AHSS) agenda – the ability of African nations to finance, manufacture and govern their own health systems and medical countermeasures.^1^ Notably, one of the AHSS’s strategic pillars calls for the digital transformation of Africa’s health system. Yet, while progress exists, the digitisation of the public health system in Africa remains fragmented, underfunded and poorly integrated.^2^ To move from pilots to scale, Africa must redefine a clear agenda. We propose five priorities to chart Africa’s digital public health future, particularly to ensure continental health security.

Firstly, each African Union (AU) member state should issue a digital health card with a unique identifier for every citizen at birth, which will be used to follow everyone across the entire lifecycle and across sectors.^3^ This identifier would connect birth registration, immunisation, school enrolment, and later adulthood health and social records, ensuring an integrated continuum of identity and care. The digital health wallet, anchored in this unique identifier, would evolve into a lifelong electronic medical record, portable across borders and essential for continuity of care in an increasingly mobile population. Beyond improving service delivery, it would serve as a critical instrument for contact tracing and cross-border surveillance, enabling real-time exchange of health information between neighbouring states, and facilitating coordinated epidemic detection and response. Such a system could overcome the pervasive challenges of fragmented paper-based registries, lost vaccination cards, and weak civil registration systems.^3,4^ It would also strengthen accountability for reaching zero-dose children, enable more efficient resource allocation, and anchor Africa’s health information ecosystem in an individual-based, rights-driven and interoperable approach. Despite their relevance, the majority of African countries have not issued unique digital identifiers, underscoring the need to strengthen the implementation of digital unique identifiers for citizens in Africa.

Secondly, the health system at community levels, including community health workers (CHWs) and primary healthcare units, must be digitised through the implementation of DHIS2 tracker (Oslo University, Oslo, Norway) for every CHW using smartphones or related gadgets. Notably, DHIS2 is an open-source, fully customisable software platform currently in use across all AU member states for routine health information management. Community health workers remain the frontline of Africa’s health systems, yet most still work with paper tools, affecting the quality and timeliness of critical health data. Equipping each CHW with a smart gadget preloaded with the DHIS2 tracker would revolutionise community-based surveillance, case detection and programme monitoring. Real-time data from villages could feed into district and national dashboards, reducing reporting delays from months to minutes. This transformation would enhance early outbreak warning, facilitate the monitoring of maternal and child health services, and strengthen accountability at the community level.^5^ Moreover, digital empowerment of CHWs would improve motivation, supervision, and integration within formal health systems.^4^ Moreover, primary healthcare facilities (health centres) in Africa continue to be hampered by paper registers and delayed reporting. Providing every health centre with a digital tool loaded with DHIS2 tracker would institutionalise electronic surveillance at the facility level. This shift would ensure that immunisation coverage, maternal health indicators, and stock management are monitored in real-time. Integration with laboratory and pharmacy modules could improve supply chain management, while feedback loops to frontline staff would foster data-driven decision-making. Importantly, digitising health centres would generate high-quality, timely data to inform local, national, and continental policy.^5,6^

Thirdly, implementing the second-generation DHIS2, integrating health programmes and emergencies, is essential in Africa. DHIS2 has become Africa’s most widely used health information system. However, most deployments are still programme-specific and siloed. Africa now requires a second generation of DHIS2, an integrated platform that combines programme monitoring with public health emergency management. Such a system would allow Ministries of Health to track human immunodeficiency virus (HIV), malaria, and maternal health alongside cholera, mpox or coronavirus disease 2019 (COVID-19) outbreaks in one platform. Standardised dashboards, harmonised indicators, and interoperability with civil registration, laboratory, and supply chain systems would improve efficiency and resilience. This next generation of DHIS2 would reflect Africa’s move from parallel systems to integrated, flexible digital architectures.

Fourthly, a continental public health data centre that can host all the health-related data at a central level (Africa Centres for Disease Control and Prevention [CDC]) and provides role-based access to member states (users) needs to be strengthened. Data sovereignty is fundamental to Africa’s future. Establishing an operational continental data centre at Africa CDC headquarters would provide a centralised, secure hub for the storage and management of Africa’s public health data. Such a facility would ensure interoperability across member states, enable comparative analytics and provide a trusted continental repository for epidemic intelligence. Importantly, the data centre would integrate with the Africa Pathogen Genomics Initiative, vaccine deployment platforms and disease surveillance systems. It would serve as the backbone for Africa’s collective capacity to anticipate, prevent and respond to health threats.

Lastly, a continental public health knowledge management hub that could facilitate real-time health knowledge exchange, grounded in evidence generated from data captured and reported by member states, needs to be strengthened. Real-time health knowledge exchange among and within member states is critical to ensure continental health security. The knowledge management hub serves as a ‘one-stop’ hub to ensure health knowledge is available, accessible and usable. This can be facilitated by artificial intelligence (AI)-powered systems that identify and archive health knowledge, synthesise and summarise it in an accessible form, and disseminate it to public health practitioners and authorities to ensure informed decisions and responses to public health emergencies.

## The enablers of digitisation

Reliable connectivity at all health facilities is the backbone of Africa’s digital health agenda. Without stable internet access and electricity at the primary point of care, digital innovations, such as telemedicine, electronic medical records, disease surveillance or supply chain tracking, cannot function effectively or equitably.^6,7^ Yet, many rural and remote clinics remain offline, leading to gaps in health data, fragmented patient care and delays in outbreak detection. Ensuring that every primary healthcare facility is connected guarantees that no community is left behind, that health workers can access decision-support tools in real-time, and that Ministries of Health can obtain a complete and accurate picture of population health. Achieving this requires Africa to mobilise strong public–private partnerships with telecommunications operators, scale up affordable satellite and mobile broadband solutions, and systematically integrate connectivity into health infrastructure investments – so that every new or rehabilitated clinic is digital-ready. Once connectivity is secured, Africa will be positioned to leapfrog towards a fully integrated digital health ecosystem that strengthens health systems and saves lives.

Artificial intelligence will be a critical enabler of Africa’s digital health agenda. Artificial intelligence-driven analytics can detect anomalies in surveillance data, predict epidemic hotspots and optimise resource allocation.^8^ Machine learning tools can support clinical decision-making at the frontline, while natural language processing can enable voice-to-text reporting for CHWs working in remote areas. Integrating AI into Africa’s digital health architecture would transform the continent’s ability to anticipate epidemics rather than react to them. However, this requires investment in digital literacy, ethical frameworks, and data governance to ensure that AI-driven solutions are equitable and rights-based.

To ensure seamless data exchange and integration across health systems, the national digital health strategy must prioritise interoperability standards. Adopting globally recognised frameworks such as Health Level 7 International–Fast Healthcare Interoperability Resources (HL7–FHIR), the International Patient Summary (IPS), and Integrating the Healthcare Enterprise (IHE) profiles is essential for enabling secure, consistent, and scalable health information exchange. These standards reduce fragmentation, promote vendor neutrality, and support real-time decision-making during emergencies. By embedding interoperability into the national architecture, countries can strengthen health system resilience, enhance patient safety, and align with international best practices for digital health transformation.

## The way forward

The digitisation of Africa’s public health is no longer a technological challenge; it is a political and financial imperative. The five priorities outlined are both feasible and urgent. Their implementation would require strong political commitment, sustainable financing, and a coordinated approach by the African Union Member States and global partners.

To avoid dependency and ensure long-term resilience, Africa must also build a business model that generates resources to sustain digital health systems. This means developing financing mechanisms that blend domestic investments, public–private partnerships, and regional innovation funds to support continuous upgrades, maintenance, and research. A self-sustaining model would not only reduce reliance on donor cycles but also create an ecosystem that incentivises African innovators, protects data sovereignty, and ensures that digital health remains adaptive to emerging technologies.

Without bold steps, Africa risks remaining dependent on fragmented donor-driven systems, missing opportunities to harness the demographic dividend, and being unprepared for the next pandemic, posing a material risk to health security. Conversely, by investing in a coherent digital health future, Africa can leapfrog traditional barriers and position itself as a global leader in digital public health innovation.
